# Important Notice

**Published:** 1910-04

**Authors:** 


IMPORTANT NOTICE.—Those of our readers who are interested in the various forms of physiologic therapeutics (including hydrotherapy, massage, electrotherapy, hyperemia, etc.), will be glad to know that it is proposed to shortly inaugurate a new journal devoted solely to the delineation of the progress made along these lines of therapeutic endeavor.

*The American Journal of Physiologic Therapeutics* will be published bimonthly and the subscription price will be $1.00 a year. The names and addresses of all interested physicians should be sent in, and those desirous of subscribing at once may enclose their remittance when writing. It is to be hoped that a widespread interest may be aroused in this matter. Write *now,* while this is fresh in your mind, to *The American Journal of Physiologic Therapeutics,* 72 Madison Street, Chicago.

